# Mechanistic understanding of iron toxicity tolerance in contrasting rice varieties from Africa: 1. Morpho-physiological and biochemical responses

**DOI:** 10.1071/FP18129

**Published:** 2018-10-29

**Authors:** Dorothy A. Onyango, Fredrickson Entila, Mathew M. Dida, Abdelbagi M. Ismail, Khady N. Drame

**Affiliations:** A Africa Rice Center (AfricaRice), 01 BP 4029, Abidjan 01, Cote d’Ivoire; B International Rice Research Institute (IRRI), DAPO Box 7777, Metro Manila, Philippines; C Department of Applied Plant Sciences, Maseno University, Private bag, Maseno, Kenya; D Present address: Biosciences of east and central Africa, PO Box 30709-00100, Nairobi-Kenya

**Keywords:** adaptation traits, biochemical mechanisms, Fe-toxicity tolerance, physiological mechanisms, rice

## Abstract

Iron (Fe) is a fundamental element involved in various plant metabolic processes. However, when Fe uptake is excessive, it becomes toxic to the plant and disrupts cellular homeostasis. The aim of this study was to determine the physiological and biochemical mechanisms underlying tolerance to Fe toxicity in contrasting rice varieties adapted to African environments. Four varieties (CK801 and Suakoko 8 (tolerant), Supa and IR64 (sensitive)) selected from our previous work were analysed in more detail, and the first part of this study reports morphological, physiological and biochemical responses induced by Fe toxicity in these four varieties. Morphological (shoot length, root length, number of lateral roots), physiological (photosynthesis rate, stomatal conductance, transpiration rate, fluorescence, relative water content and cell membrane stability) and biochemical (tissue Fe, chlorophyll pigments, soluble sugars, protein and starch) traits were measured, as appropriate, on both shoot and root tissues and at different time points during the stress period. Fe toxicity significantly (P ≤ 0.05) reduced growth and metabolism of all the four varieties. Tolerant varieties showed more lateral roots than the sensitive ones, under Fe toxic conditions as well as higher photosynthesis rate, chlorophyll content and cell membrane stability. Strong dilution of Fe concentration in cells was identified, as one of the additional tolerance mechanisms used by CK801, whereas Suakoko 8 mainly used strong mobilisation of carbohydrates at the early stage of the stress period to anticipate metabolite shortage. Traits associated with Fe toxicity tolerance in this study could be specifically targeted in trait-based breeding programs of superior lowland rice varieties tolerant of Fe toxicity.

## Introduction

Iron plays pivotal functions in plant respiration, cell division, synthesis of chlorophyll, and electron transport chain of photosynthesis (Müller *et al*. 2015). However, Fe^2+^ also acts as a catalyst in Fenton reactions, producing reactive oxygen species (ROS) that are potentially harmful to the cell and that induce oxidative damage in plants (Kao *et al*. 2001; Onaga *et al*. 2016). Therefore, when Fe is absorbed in excess by plants, it can shift the cell redox balance towards a pro-oxidant state, causing alterations in the morphological, biochemical and physiological characteristics of the plant (Hell and Stephan 2003) and in the most severe cases, lead to plant death. To maintain balanced levels of Fe that enable optimal plant cell functions while avoiding the harmful effects of Fe-induced oxidative damages, plants must establish tight control mechanisms of cellular Fe levels.

Rice plants can absorb Fe both in the divalent (Fe^2+^) and trivalent (Fe^3+^) forms. However, under aerobic conditions, the dominant Fe^3+^ form is often complexed in the soil and less available to plants. In flooded conditions, as it is the case in lowland rice growing environments, the reduction of Fe^3+^ into Fe^2+^ is favoured, especially under acidic and low redox conditions and the Fe^2+^ form is soluble. Hence, lowland rice cultivated in Fe rich environments under constant or partial flooding, without good water management, faces the risk of being exposed to Fe toxicity at any stage of growth. When rice plants experience Fe toxicity, they usually develop orange brown spots on the leaves. This symptom is commonly called leaf bronzing and it is thought to occur as a result of oxidative stress (Apel and Hirt 2004). In addition, strong growth depression and damaged root systems can be observed on rice plants grown under excess Fe conditions.

As evidenced by many studies, rice plants under Fe toxicity stress display a wide range of responses as part of their strategies to overcome the stress. These strategies include both avoidance and tolerance mechanisms and their efficiency may vary with the type of Fe toxicity occurring in the growth environment, its duration and intensity (Becker and Asch 2005; Frei *et al*. 2016). Studies conducted by Dufey *et al*. (2009) reported that in severe cases of iron toxicity, rice plants showed reduction of the water content, and chlorophyll content index and an increase in the stomatal resistance, shoot iron uptake and iron concentration of the roots and shoots. Stomatal resistance has also been found to be positively correlated with leaf bronzing index in iron toxic conditions (Dufey *et al*. 2009). According to Goicoechea *et al*. (2001) a rice plant closes its stomata in order to limit the transpiration rate, which may affect the CO^2^ exchange rate and related photosynthetic functions, meaning that plant growth would be reduced. However, in the case of the genotypes used in the study conducted by Dufey *et al*. (2009), stomatal resistance was observed to be a late mechanism of survival after a high iron concentration had been reached in the leaf tissue, since the more sensitive plants presented both higher stomatal resistance and high iron concentration. Several studies have also reported a reduction in chlorophyll pigments in association with stomatal closure, leading to a reduction in photosynthesis (Dingkuhn *et al*. 1999; Sairam and Saxena 2000; Awal and Ikeda 2002). Such complexity may explain why mainly minor quantitative trait loci (QTL) were identified for Fe toxicity tolerance (Dufey *et al*. 2009; Wu *et al*. 2014; Onaga *et al*. 2016). Although several reactions and cellular adjustments are required to circumvent the harmful effects of Fe toxicity, most of the genetic studies on this stress have looked at tolerance traits at the plant level (e.g. leaf bronzing, plant height, biomass). It is indeed challenging to evaluate traits at a tissue or cellular level on large populations. However, understanding the specific traits contributing to Fe toxicity tolerance in potential donors and singling out individual factors that contribute to these traits could be a first step towards more tailored mapping studies and greater successes in rice improvement for diverse Fe-toxic environments.

In this study four varieties contrasting in Fe toxicity tolerance (including two potential donors) were studied for various physiological, biochemical and anatomical traits with the aim of gaining a holistic perception of traits underlying rice tolerance mechanisms to Fe toxicity and recommend breeding target traits. This work focuses on the first part of the study, which investigated morphological, physiological and biochemical changes induced by Fe toxicity over time and traits associated with Fe toxicity tolerance.

## Materials and methods

### Plant material

Four rice varieties were used in this study (Table 1). Seeds of all varieties were obtained from AfricaRice. All the varieties tested are adapted to lowland ecology and belong to *Oryza sativa* L. ssp. *indica*. Previous studies reported that IR64 is moderately sensitive (Wu *et al*. 1997; Dufey *et al*. 2012). Suakoko 8 is well known for its tolerance to Fe toxicity (Audebert and Sahrawat 2000; Sikirou *et al*. 2015) and Supa was reported as sensitive to Fe toxicity (Onaga *et al*. 2013). The other variety CK801 was selected based on preliminary experiments (KN Drame, unpubl. data) and recommendations of the national Agricultural Research Institute of Guinea (IRAG).

**Table 1:** Effects of iron treatment (Fe), variety (Var), growth stage (time) and their interactions on the variation of relevant plant traits measured in this study and average performance of plants grown under control and excess iron conditions over the duration of the growth period (3 weeks)

	Iron treatment	Time	Variety	Fe × time	Time × Var	Fe × Var	Fe × time × Var	Control means	Stress means
*Morphological traits*															
Shoot length	**	***	****	***	***	****	****	50.32	49.2
Root length	**	**	***	*	**	****	****	23.9	20.6
No. of lateral roots	***	***	**	***	**	***	***	4.3	6.9
*Physiological traits*															
Photosynthesis	**	*	*	*	*	**	**	17.5	8
Cellular CO^2^	*	**	ns	*	ns	*	*	339.4	299.4
Transpiration	**	ns	**	**	*	*	ns	5.3	2.5
Conductance	****	***	ns	**	ns	ns	ns	543.7	206.9
Fluorescence	**	**	*	**	*	*	**	494.8	960.3
Relative water content	***	na	***	na	na	***	na	81.6	58.7
Cell membrane stability	***	na	***	na	na	***	na	86.7	74.1
*Biochemical traits*																
Soluble sugars	****	****	****	****	****	****	****	62.2	51.9
Proteins	****	****	****	****	****	****	****	0.3	0.1
Starch	****	****	****	****	****	****	****	18.7	21

**Fig. 1:**
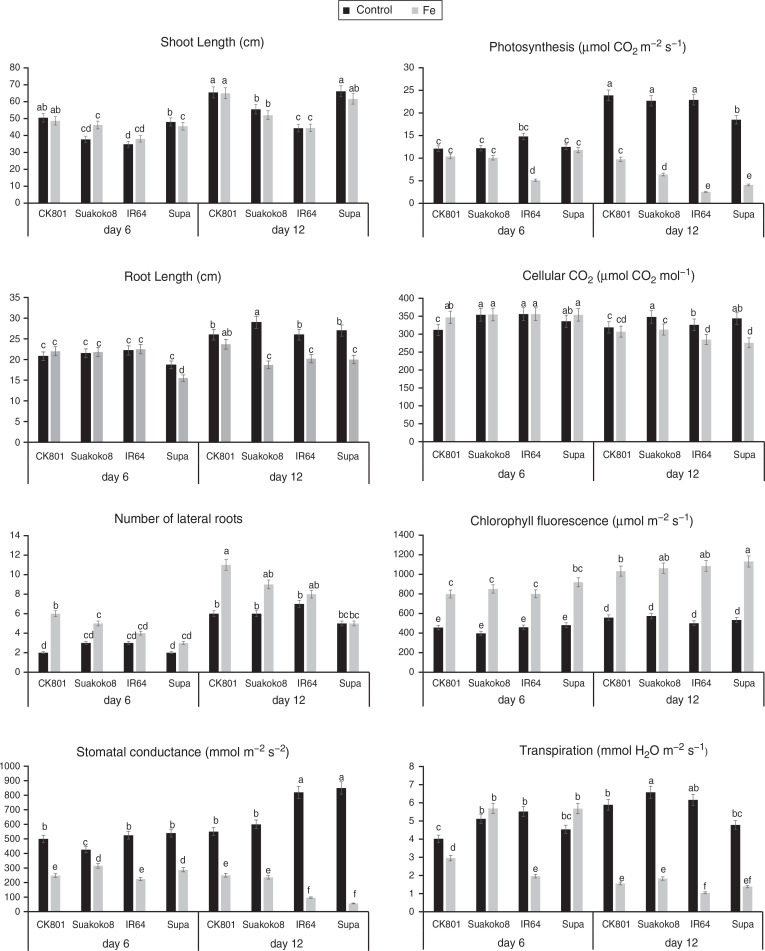
Differential morphological and physiological responses of the four varieties studied depending on the iron treatment (control and excess Fe) and stress duration (after 6 days and 12 days of stress).

**Fig. 2:**
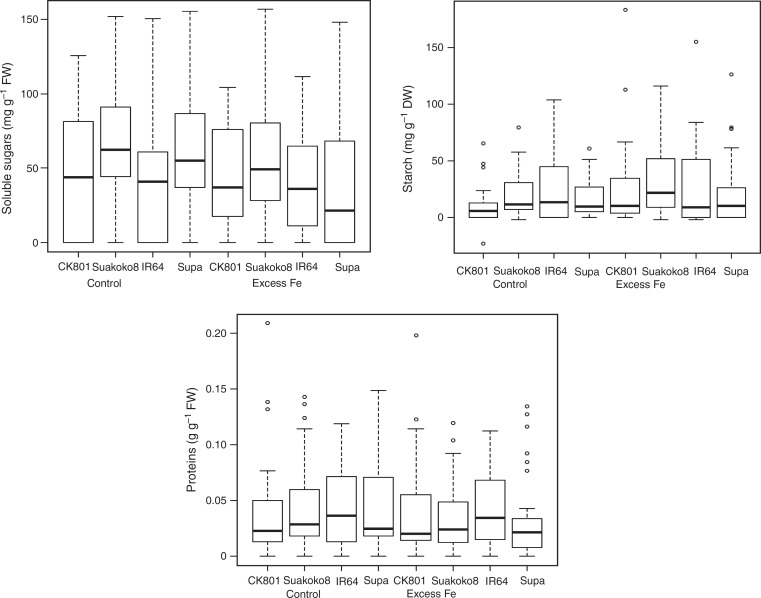
Variation of biochemical traits (soluble sugars, starch and proteins) in four varieties contrasting for iron toxicity tolerance grown under control and excess iron (Fe) conditions.

Units of measurement are the same as in Figures 1 and 2. Statistically significant differences are indicated: ****, *P* ≤ 0.0001; ***, *P* ≤ 0.001; **, *P* ≤ 0.01; *, *P* ≤ 0.05; ns = not significant; na = not applicable

### Optimised hydroponic culture system and plant growth

When Fe concentration is high in a solution it tends to precipitate with other elements, especially phosphorus (P), resulting in a low availability of both elements. In order to minimise such precipitation, different compositions of Yoshida (Yoshida 1976) and Magnavaca (Magnavaca *et al*. 1987) nutrient solutions were tested in Fe toxic conditions using Geochem software (Shaff *et al*. 2010). The most stable solution was obtained with optimised Magnavaca nutrient solution (Magnavaca *et al*. 1987; Famoso *et al*. 2010) composed as follows: 1.65 mM Mg (MgSO_4_.7H_2_O), 1 mM Ca (CaCl_2_.2H_2_O), 0.05 mM P (NaH_2_PO_4_.H_2_O), 0.56 mM K (K_2_SO_4_), 1.43 mM N (NH_4_NO_3_), 0.08 mM Fe^3+^ (HEDTA-Fe), 0.012 µM Mn (MnCl_2_.4H_2_O), 0.033 µM B (H_3_BO_3_), 0.003 µM Zn (ZnSO_4_.7H_2_O), 0.0008 µM Cu (CuSO_4_.5H_2_O), 0.001 µM Mo (Na_2_MoO_4_.2H_2_O), 0.016 mM Si (Na2SiO_3_.9H_2_O), 5.37 mM Fe^2+^ (FeSO_4_.7H_2_O). Available Fe^2+^ and P concentrations in the nutrient solution were confirmed by daily measurement for 15 days using ferrozine and molybdate assay respectively. In brief, a sample of culture solution was cleared by centrifugation at 3220g for 5 min and 20 mL was mixed with 600 mL of 1 mM ferrozine. Absorbance at 562 nm was measured and concentration of Fe^2+^ in mM was calculated according to Stookey (1970) using the following formula: [Fe^2+^]= A^562^/(27900 × DF × 10^3^) where A^562^ is the absorbance at 562nm and DF is the dilution factor. To determine available P, a sample of the cleared solution was mixed with 0.1 mM sulfuric-ammonium molybdate at equal volumes and absorbance at 882 nm measured. Concentration of P in the sample was determined using a standard curve plotted using known concentrations of P (0, 0.5, 1, 2, 5, 10, 50 and 100 mM in optimised Magnavaca solution).

All rice seeds were incubated at 50°C for 5 days to break possible seed dormancy. They were surface-sterilised by soaking in 70% ethanol for 10 min and thereafter thoroughly rinsed with de-ionised water. Then, the seeds were soaked in de-ionised water for 48 h at 32°C and germinated on wet paper towel rolls at 32°C for another 48h. At the end of the incubation period, the paper rolls holding the seedlings were transferred to the screenhouse to acclimatise at ambient conditions. The following day, the seedlings were transferred to culture trays containing the optimised Magnavaca nutrient solution (Famoso *et al*. 2010). Seedlings were allowed to grow for 2 weeks before excess Fe (5.4 mM Fe^2+^ added as FeSO_4_•7H_2_O) was applied to half of the trays. The other half was used as control. The pH of the culture solution as well as the concentrations of P and Fe^2+^ were monitored daily as described above and adjusted to their respective initial values. The culture solution was renewed every 5 days. The experiments were set up in a split-plot with randomised complete block design.

### Morphological and physiological measurements

All morphological and physiological parameters were measured at the beginning of the stress treatment and thereafter, once a week, except the destructive measurements which were done only at the end of the experiment. Morphological traits included shoot length, root length and number of lateral roots. Shoot length and root length were measured from the culm base to the tip of the longest leaf and from the culm base to the tip of the longest root, respectively. The number of functional lateral roots was counted on each plant. After 16 days of Fe treatment, leaf bronzing was scored on the basis of a visual assessment of Fe toxicity on the whole plant following the Standard Evaluation System (SES) (IRRI 2002). Physiological parameters included chlorophyll fluorescence (expressed as electron transport rate, ETR), leaf stomatal conductance, relative water content (RWC), content of chlorophyll pigments and carotenoids and membrane stability. Gas exchange measurements were done on the youngest fully expanded leaf on three plants per variety per replication and per treatment between 0900 and 1300 hours.

Chlorophyll fluorescence was monitored using the pulse amplitude modulation (MINI-PAM, Walz). Leaf stomatal conductance was measured using an automatic porometer (AP 4, Delta-T Devices). Cellular CO_2_, transpiration, and photosynthesis rates were measured using a portable photosynthesis system (LI6400XT, LI-COR Biosciences) following the manual’s guidelines. Internal parameters were fixed at a photosynthetic photon flux density (PPFD) of 1200 µmol m^—2^ s^—1^ by using a LED light source at a reference CO^2^ concentration of 400 ppm. For the quantification of photosynthetic pigments (chlorophyll *a* (chl*a*), chlorophyll *b* (chl*b*) and carotenoids), leaf samples were collected and freeze-dried for 3 days. The dry weight of the samples was determined and the samples placed in 15 mL vials covered with aluminium foil to protect the extracts from the light. To these vials, 10 mL of 95% ethanol was added and samples were incubated in the dark for 24 h. Absorbance was measured at different wavelengths and chlorophyll and carotenoid concentrations were calculated according to Lichtenthaler and Buschmann (2001):

$$Chlorophyll\;a\;\left(C_{a}\right)\left(\mu \;g\;mL^{-1}\right)=\left(13.36\;\times \;A_{664.1}\right)\;-\left(5.19\;\times \;A_{648.6}\right),$$ (1)

$$Chlorophyll\;b\;\left(C_{b}\right)\left(\mu \;g\;mL^{-1}\right)=\left(27.43\;\times \;A_{648.6}\right)\;-\;\left(8.12\;\times \;A_{664.1}\right),$$ (2)

$$Carotenoid\left(C_{X+c}\right)\left(\mu g/mL^{-1}\right)=\left[\left(1000\;\times \;A_{470}-2.13\;\times \;C_{a}\right)-97.64\;\times \;C_{b}\right]/209,$$ (3)

$$Total\;chlorophyll\;content\;=\;\mu \;g\left(C_{a}+C_{b}\right)/g\;DW.$$ (4)

Damage to the photosynthetic apparatus was determined as the ratio between total chlorophyll and carotenoid contents: (C_a_ + C_b_)/(C_x+c_). Membrane stability was assessed using the electrolyte leakage test. Fifteen leaf discs were freshly cut (0.5 cm^2^ each) and rinsed three times (2–3 min) with distilled water to remove ions on the cut surfaces and rinsing was done gently to avoid mechanical damage to the leaf that would increase the amount of ion leakage. Leaf discs were then floated on 10 mL of distilled water for 16 h at room temperature under constant shaking. Ion leakage in the solution was measured using a conductivity meter. The leaf discs were then incubated in an oven (90°C) for 2 h and the conductivity measured. The percentage of cell membrane stability was expressed as (100 – ((EC_0_/EC_f_) × 100)), where EC_0_ and EC_f_ are the conductivity before and after heating respectively (Agarie *et al*. 1995). To determine RWC, fully expanded leaves were sampled and immediately weighed to determine the FW. Leaves were then immersed in Petri dishes containing 5 mM CaCl_2_ overnight at room temperature in a closed box to reduce vapour loss. The next day, leaves were removed and quickly blot-dried on a tissue paper then weighed to determine turgid weight (TW). DW was determined after oven-drying the leaves at 74°C for 3 days. The RWC of the leaf was calculated using the formula RWC = ((FW – DW)/ (TW – DW)) × 100.

### Biochemical analyses

Biochemical analyses were done on root and shoot samples collected at five different time points during the stress period, with 4-day interval between two samplings. The sampling scheme was as follows: sampling point 1 = just before excess Fe application; sampling point 2 = 4 days after the application of Fe treatment; sampling point 3 = 8 days after; sampling point 4 = 12 days after; and sampling point 5 = 16 days after application (end of experiment). All samples were snap-frozen in liquid nitrogen immediately after collection and transferred to —80°C. Later, the samples were ground to fine powder using liquid nitrogen, making sure to avoid their thawing. The ground samples were weighed and used for the various analyses as follows.

### Soluble sugars

Samples (200 mg) of ground shoot and root were mixed with 7 mL of 80% ethanol, vortexed at maximum speed for 2 s and centrifuged at 2465g for 5 min. The supernatant was collected and the process was repeated twice, adding 3 mL of 80% ethanol the third time. The supernatant was adjusted to 25 mL using 80% ethanol and the container covered with parafilm to avoid evaporation (Fales 1951). A sample assay was prepared with different proportions of the shoot and root samples extracts. For root samples, 0.1 mL of extract plus 1 mL of anthrone were used whereas for shoot samples, 0.05 mL of sample, plus 0.05 mL of 80% ethanol and 1 mL of anthrone were used. All the samples were incubated in a water bath at 95°C for 10 min and the reaction stopped in an ice bath. All samples were vortexed at maximum speed for 2 s before absorbance readings were taken at 620 nm using SPECTROstar Nano (BMG Labtech). Total soluble sugars were determined by plotting absorbance readings on a standard curve obtained with different concentrations of glucose and expressed as the amount of sugar per unit DW of tissue sample used in the extraction.

### Starch content

Dry residues from the sugar assay were weighed together with 10 mg of starch standard and they were mixed with 2 mL of acetate buffer. All the samples were incubated in a water bath for 3 h at 95°C with regular mixing, every 20 min. The mixture was cooled to 55°C and 1 mL of amyloglucosidase in acetate buffer was added immediately. The samples were vortexed at maximum speed for 2 s and incubated for 24 h at 37°C, after which the samples were centrifuged at 2465g for 10 min and the supernatant transferred to a 25-mL volumetric flask. Solid residues were cleaned using 3 mL of Nano pure water vortexed and centrifuged at 2465g for 10 min. The supernatant was added to the previous one and the extract volume was toped up to 25 mL using Nano pure water. The starch sample was used to prepare the standards. The sample assay was prepared by mixing 0.6 mL of the extract with 3 mL of peroxidase–glucose oxidase/odianisidine (PGO) enzyme colour reagent, vortexing the mix at maximum speed and incubating it in the dark for 30 min (Draeger *et al*. 1988). Samples absorbances at 450 nm were determined using SPECTROstar Nano (BMG Labtech). Starch content was expressed per unit DW of tissue sample used in the extraction.

### Protein content

Samples (200 mg) of finely ground shoot and root tissues were kept on ice to inactivate any enzyme and 2 mL of cold extraction buffer (100 mM HEPES-KOH, pH8.0) was added immediately before the samples thaw. The samples were vortexed at maximum speed for 2 s, agitated at 4°C for 10 min and centrifuged at 10 000g, 4°C for 10 min. The crude extract was collected and the residue discarded (Tomlinson *et al*. 2004). This crude extract was then subjected to dialysis for 24 h, with constant agitation at 4°C and changing the dialysate every 6 h to ensure maximum purification of the proteins. Purified proteins were quantified following Bradford (1976).

In addition, to these traits, tissue Fe content was determined at the end of the experiment from the four uppermost and the three lower leaves of the harvested plants. The leaves were detached from the roots at the culm base and weighed to determine FW. Roots were rinsed thoroughly using distilled water, blot-dried on tissue paper, and their FW determined. The fresh roots were incubated for 3 h at 25°C in 45 mL of a solution (pH 6.5) containing 0.27 M sodium citrate (Na_3_C_6_H_5_O_7•_2H_2_O), 0.11 M sodium bicarbonate (NaHCO_3_) and 3 g of sodium dithionite (Na_2_O_4_S_2_), to remove the Fe plaques on the roots. The washed roots and shoots were oven-dried at 70°C for 3 days and the DW determined. The dried samples were finely ground in a stainless electric grinder. Subsamples of 300 mg of ground samples were ashed in a furnace at a temperature of 325°C until all smoke dissipated. Complete ashing was then done overnight at 490°C. The samples were cooled at room temperature and the ashes suspended in 50 mL of 1N HCl. The dissolved ashes were centrifuged at 10 000g for 10 min and intracellular Fe determined in the extracts using an atomic absorption spectrophotometer (Shimadzu, model AA60IFG).

### Statistical analysis

Statistical analysis of the data was done using the Statistical Tool for Agricultural Research (STAR) (IRRI 2013) and R i386 ver. 3.2.2 (The R Foundation for Statistical Computing Platform, 2015). Analysis of variance (ANOVA) was performed using the general linear model (GLM) procedure. Treatment means were separated using Duncan’s multiple range test (DMRT) at P ≤ 0.05.

### Results

Effects of treatments on the variation of the plant traits measured

For all the morphological, physiological and biochemical traits studied, the effect of Fe treatment was significant (P ≤ 0.05) to highly significant (P ≤ 0.0001). The effect of time was significant (P ≤ 0.05) to highly significant (P ≤ 0.0001) for all the traits except transpiration, although variety effect was not significant for cellular CO^2^ and conductance (Table 1).

For morphological traits, the interaction between Fe treatment and time (Fe × time), time and variety (time × Var), Fe treatment and variety (Fe × Var) and between Fe treatment, time and variety (Fe × time × Var) was significant (P ≤ 0.05) to highly significant (P ≤ 0.0001) for all the traits measured. For physiological traits, the effect of Fe × time interaction was significant (P ≤ 0.05 to P ≤ 0.01) on the variation of all the traits whereas the effect of time × Var interaction was only significant at P ≤ 0.05 and not significant for cellular CO^2^ and conductance. The effect of Fe × Var interaction was significant (P ≤ 0.05) to highly significant (P ≤ 0.001) for all the traits except conductance. The interaction Fe × time × Var had a significant effect (P ≤ 0.05 to P ≤ 0.01) on trait variation except for transpiration and conductance. For the biochemical traits, all interactions had highly significant effect (P ≤ 0.0001) on trait variations (Table 1).

Over the growth period in this experiment (3 weeks), overall performances of plants subjected to excess Fe were reduced compared with the control plants, except for chlorophyll fluorescence, lateral roots and starch content. Reduction of growth traits varied between 11.1% (root length) and 15.4% (shoot length) whereas metabolic traits reduced by 16.5% (soluble sugars) and 60% (proteins). The highest reduction among physiological traits was observed for conductance (61.9%), transpiration (53%) and photosynthesis (50.2%) (Table 1).

### Morphological and physiological responses of the varieties depending on Fe treatment and stress duration

At moderate (after 6 days of exposure to excess Fe) and severe (after 12 days of exposure to excess Fe) stress levels, CK801 and IR64 maintained more or less the same shoot length in the stressed plants as in the control plants. Under moderate stress, Suakoko8 slightly boosted plant growth compared with control plants but under severe stress, a slight decrease was noted. Shoot length in Supa decreased under both moderate and severe stress levels (Fig. 1). Similarly, CK801, IR64 and Suakoko8 showed minor difference of root length between stressed and control plants at moderate stress level whereas in Supa, a decrease of root length was noted in stressed plants. At severe stress level, decrease of root length is observed in all the varieties under excess Fe conditions. The number of new lateral roots, under both moderate and severe stress levels, increased in stressed plants of all the varieties compared with control plants. The largest increase of the number of lateral roots and the highest number under excess Fe conditions were observed in CK801 followed by Suakoko8, then IR64 and Supa (Fig. 1).

Concerning physiological traits, a reduction of photosynthesis rate was observed in all the varieties under both moderate and severe stress conditions with the decrease being much more pronounced after 12 days of exposure to excess Fe than after 6 days (Fig. 1). The strongest reduction and the lowest photosynthesis rate under excess Fe conditions was observed in IR64. Under severe stress conditions, CK801 showed the highest photosynthesis rate among the four varieties. For cellular CO_2_, there was no difference between stressed and control plants of IR64 and Suakoko8 at moderate stress level. In the same conditions, a slight increase of cellular CO_2_ was observed in CK801 and Supa. At severe stress level, a decrease of cellular CO_2_ was observed in all the varieties with CK801 showing the smallest variation between stressed and control plants (Fig. 1). Chlorophyll fluorescence increased under both moderate and severe stress levels in all the varieties with the values under severe stress being higher than in moderate stress. Under excess Fe conditions, CK801 presented the lowest chlorophyll fluorescence whereas Supa showed the highest (Fig. 1).

Although the interaction Fe × time × Var was not significant for transpiration and conductance, differential varieties’ responses were also observed for these traits. At moderate stress level, transpiration significantly decreased in stressed plants of CK801 and IR64 compared with control plants whereas in Suakoko8 and Supa higher transpiration rate was observed in stressed plants. Transpiration rate decreased in all varieties under severe stress level. Stomatal conductance decreased at both moderate and severe stress levels in all the varieties but with different magnitudes (Fig. 1).

In addition, CK801 presented the healthiest plants under excess Fe conditions with an overall leaf bronzing score (LBS) of 3 and higher relative water content and chlorophyll content than the other varieties (Table 2). It was followed by Suakoko8 which recorded similar level of membrane stability than CK801 despite a higher LBS. IR64 and Supa presented comparable levels of leaf bronzing and chlorophyll content but IR64 had higher water content (within the same range as Suakoko8) than Supa. Suakoko8 recorded the highest carotenoid content followed by Supa and CK801, and lastly IR64 (Table 2).

**Table 2:** Varietal differences in plant traits measured only at the end of the experiment (relative water content, membrane stability, content of photosynthetic pigments) and only on stressed plants (leaf bronzing and iron concentration)

	RWC (%)		MS (%)		Chla		Chlb		Car		LBS	Root		LowLeaf	UpLeaf	Shoot
					(µgmL^–1^)		(µgmL^–1^)		(µgmL^–1^)			(µmol g^–1^)		(µmol g^–1^)	(µmol g^–1^)	(µmol g^–^
Treatment	Fe	Control	Fe	Control	Fe	Control	Fe	Control	Fe l	Control	Fe	Fe	Control	Fe	Fe	Control
CK801	81.4a	87.8a	85.4a	88.7a	41a	45.4a	32.3a	42.4a	8.5ab	7.9b	3	1.35a	0.04b	0.38bc	3.88b	0.02b
Suakoko 8	58.2b	85.1a	85.7a	87.4a	34.4b	43.9a	18.7b	46.1a	9.7a	4.1d	5	0.42c	0.05a	0.42b	1.1c	0.03a
IR64	56.2b	87.6a	59.2b	86.1a	20.7c	39.9b	10.3c	26.1b	5.8c	9.4a	7	0.61bc	0.04b	0.41b	3.33b	0.01c
Supa	38.3c	85.8a	66.2b	84.5a	19.9c	43.9a	9.6c	41.9a	8.2ab	6.1c	7	0.9b	0.03c	0.59a	8a	0.02b

Abbreviations: RWC, relative water content; MS, membrane stability; Chl*a*, chlorophyll *a*; Chl*b*, chlorophyll *b*; Car, carotenoids; LBS, leaf bronzing score; root, iron concentration in root tissues of excess Fe treated and control plants; LowLeaf Fe, iron concentration in lower leaves; UpLeaf Fe, iron concentration in upper leaves: shoot, iron concentration in shoot of control plants. Within columns, different letters indicate significantly different means

For all the varieties, tissue Fe concentrations in the upper leaves were higher than in the lower leaves and in the roots (Table 2). Across tissues, the highest Fe concentration was recorded in Supa, followed by CK801 and IR64. Suakoko8 presented the lowest upper-leaf and root Fe concentrations among the varieties. Supa recorded the highest tissue Fe concentrations in the lower leaves and the upper leaves. The highest inner root Fe was recorded by CK801, followed by Supa, IR64 and Suakoko8 (Table 2).

### Biochemical responses of the varieties depending on Fe treatment, time and tissues

Variation patterns of biochemical traits were significantly different across Fe treatments, sampling times and varieties. In Fig. 2, overall variation of soluble sugars, starch and proteins by Fe treatment and variety were presented. It appeared that the amount of soluble sugars in stressed plants were lower than in control plants, independent of the variety. However, Suakoko8 accumulated the highest amount of soluble sugars under both Fe treatments compared with the other three varieties. Supa, which recorded the second highest amount of soluble sugars under control conditions, became the lowest under excess Fe conditions. In both Fe treatments, the amounts of soluble sugars in CK801 and IR64 were comparable. For starch, only small variations were observed between Fe treatments and between varieties except in Suakoko8. This variety accumulated significantly higher amounts of starch in stressed plants than in control plants and much more than the other varieties in any of the Fe treatments. For proteins as well, there were very little changes between Fe treatments. However, comparison between varieties showed that IR 64 recorded the highest amounts of proteins in both control and excess Fe treatments (Fig. 2).

**Fig. 3:**
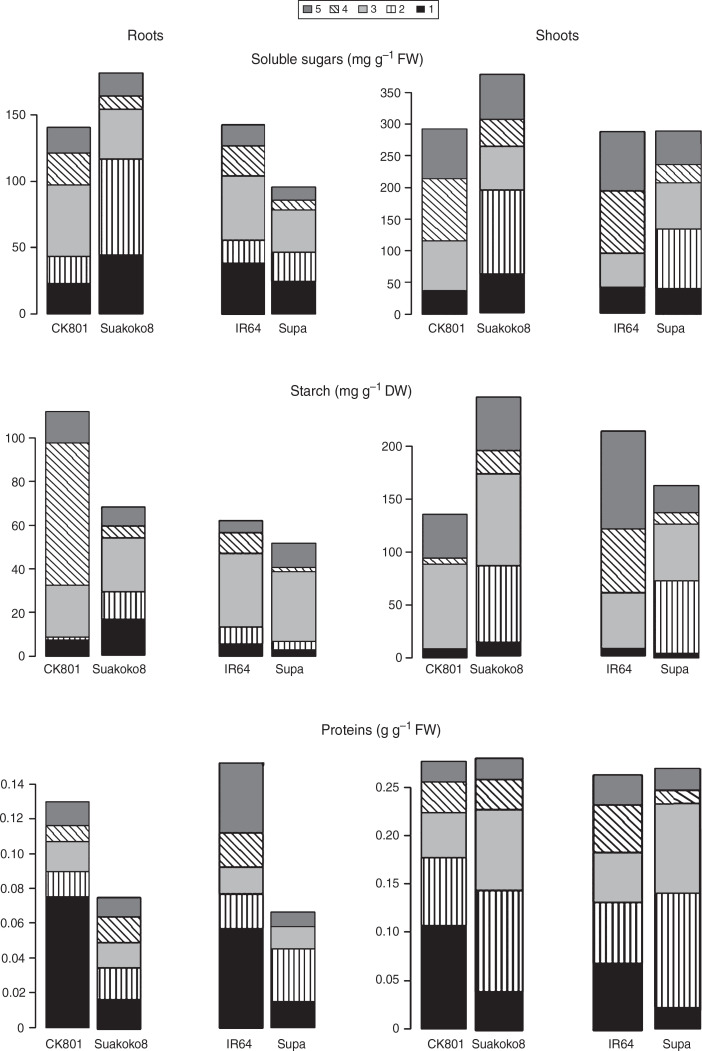
Temporal variation of biochemical traits (soluble sugars, starch and proteins) in the root and shoot tissues of plants grown under excess iron (Fe) conditions during the stress period. (1 = 0 days, 2 = 4 days, 3 = 8 days, 4 = 12 days, 5 = 16 days of excess iron treatment).

In Fig. 3, tissue-specific variations in soluble sugars, starch and proteins in response to excess Fe were presented at the different sampling points for each variety. For all the varieties, the amounts of soluble sugars, starch and proteins present in the roots were lower than in the shoots. On average, Suakoko8 recorded the highest amount of soluble sugars in both the roots and shoots, whereas Supa presented the lowest values and CK801’s sugar levels were comparable with IR64 in both tissues. In the roots, average amount of starch in CK801 was the highest among the varieties whereas that of Supa was the lowest. In the shoots, average amount of starch in IR64 was the highest, followed by Suakoko8 and the average starch level in CK801 was within the same range as in Supa. For protein contents in the roots, IR64 recorded, on average, the highest amount of proteins followed by CK801, Supa and Suakoko8. In the shoots, tolerant varieties Suakoko8 and CK801 had lightly higher protein contents than IR64 and Supa, which were comparable.

Concerning varietal differences at the different time points, it was noted that at sampling point 1 (prior to plant exposure to excess Fe), Suakoko8 recorded the highest amounts of soluble sugars and starch in both roots and shoots, whereas CK801 recorded the highest protein content in both tissues. At sampling point 2 (after 4 days of exposure to excess Fe), the protein and sugar levels in Suakoko8 were still the highest in both tissues and its protein content was the highest in the shoots. In the roots it was Supa which recorded the highest amount of proteins. Starch and soluble sugars in the shoots of CK801 and IR64 could not be measured at this sampling point. At sampling point 3 (after 8 days of exposure to excess Fe), CK801 recorded the highest amounts of soluble sugars in both root and shoot tissues. IR64 and Supa presented the highest amounts of starch in the roots whereas in the shoots, it was CK801. CK801 also recorded the highest level of proteins in the roots whereas in the shoots, the highest amount of proteins was observed in Suakoko8 and Supa. At sampling point 4 (after 12 days of exposure to excess Fe), CK801 and IR64 recorded the highest amounts of soluble sugars in both root and shoot tissues. CK801 also showed the highest starch content in the roots whereas in the shoots it was IR64. For protein contents, IR64 outperformed the other varieties in both tissues. At sampling point 5 (after 16 days of exposure to excess Fe), CK801 and IR64 recorded the highest amounts of soluble sugars and starch respectively in the roots and in the shoots. As well, IR64 recorded the highest amount of proteins in both tissues (Fig. 3).

### Discussion

Previous studies have reported differential responses of rice varieties under Fe-toxicity. However, in the present work we have presented one of the rare time course studies of Fe-toxicity induced responses (up to five time points) in different tissues (roots and shoots) and with varieties of different tolerance level (tolerant, moderately sensitive and highly sensitive). Further, care was taken to undertake this study with varieties whose tolerance levels in nutrient culture solution had been previously confirmed in the field in various studies, to ensure that selected tolerance traits would also express in natural field conditions. The tolerant varieties used in this study, CK801 and Suakoko 8, were both selected at known Fe toxicity hotspot sites, respectively at Kilissi (Guinea) and Suakoko (Liberia) and Suakoko 8 has been extensively used as a tolerant check in different studies (Dramé *et al*. 2011; Sikirou *et al*. 2015, 2016).

Analyses of varieties’ performances depending on Fe treatment and stress duration revealed differential responses between the varieties and within the same tolerance group, in few traits. In response to toxic Fe concentrations, rice varieties, irrespective of their tolerance levels, actively altered their morphological, physiological and biochemical attributes to overcome the stress and limit damages.

### Morphological and physiological responses

In all varieties, a reduction in root length was observed as the stress increased, confirming root growth inhibition as one of the negative effects of Fe toxicity (Li *et al*. 2015). Other reports have shown that roots are considerably damaged by Fe toxicity and usually they become short, coarse, blunted, and dark brown in colour (Genon *et al*. 1994). However, in the present study, the tolerant varieties formed new roots despite the stress as opposed to the sensitive varieties. This was viewed as a mechanism used by the tolerant varieties to enable them to continuously absorb nutrients to support growth and avoid the possible effects that could result from nutrient precipitation on the active root nutrient uptake sites. Kang *et al*. (2011) showed the formation of lateral roots in tolerant NERICA sister lines and suggested that it could be closely associated with reducing Fe absorption. A similar phenomenon was also reported in other rice varieties screened for Fe toxicity tolerance (Engel 2009). In addition, in the case of CK801, strong biomass production under stress, as evidenced by high shoot growth was observed. These findings agree with those of Rout *et al*. (2014) who reported increased shoot growth in tolerant rice cultivars under Fe-toxic environments. Production of a large volume of shoot decreases Fe toxicity effects through Fe dilution (Audebert and Fofana 2009). Further, CK801 recorded the highest relative water content in its tissues which may lower the internal concentration of Fe further.

Becker and Asch (2005) explained that translocation of Fe in the xylem follows the transpiration stream. Hence, stomatal closure could be a mechanism of avoidance in order to limit the uptake and translocation of Fe in the aerial parts. Low stomatal conductance under Fe toxic conditions was reported in different studies (Dufey *et al*. 2009; Engel 2009; Stein *et al*. 2009, 2014). However, in the present study, both tolerant and sensitive varieties significantly reduced their stomatal conductance from the first week of Fe treatment, suggesting that this mechanism may be a common reaction in response to Fe toxicity. A very low stomatal conductance and almost null transpiration rate were observed respectively in Supa and IR64, in the second week of stress. Such extreme reactions could be more harmful than beneficial because of the immediate consequences on gas exchanges and photosynthesis. Reports have shown that limiting transpiration rate may affect CO_2_ exchange rate and related photosynthetic functions, resulting in reduced plant growth (Goicoechea *et al*. 2001). Therefore, the difference between tolerant and sensitive varieties may reside in their capacity to balance efficiently stomatal closure and transpiration without drastically compromising gas fluxes through the stomata. This was evidenced by the high CO_2_ concentrations and correlated high photosynthesis rates observed in the tolerant varieties CK801 and Suakoko8, compared with the sensitive varieties as well as their high levels of both chlorophyll a and b pigments. Several studies have reported reduction in chlorophyll pigment as being associated with stomatal closure, leading to reduced photosynthesis (Dingkuhn *et al*. 1999; Awal and Ikeda ; Sairam and Saxena 2000).

According to Suh *et al*. (2002), excess Fe in the plant gives rise to a significant increase in Cytochrome *b*_6_/f content of thylakoids, which results in a higher susceptibility of PSII to photoinhibition and, consequently, lower photosynthetic rate and higher rate of singlet oxygen (^1^O_2_) production. Similarly, Kampfenkel *et al*. (1995) reported that excessive amounts of Fe led to photo-inhibition, increased reduction of PSII, and higher thylakoid energisation in *Nicotiana plumbaginifolia* cuttings exposed to excess Fe. This could explain why chlorophyll fluorescence increased with stress duration in all the varieties, although lower levels were observed in tolerant varieties compared with sensitive varieties. In addition, rising fluorescence could be related to the high dissipation of internal energy generated from the excitation of electrons as a result of damage caused by Fe toxicity on the photosynthetic apparatus. Inefficient energy transfer in the photosystems can lead to the production of ROS as a result of electron leakage from the Fe-S centres of PSI and reduced ferredoxin (Mittler *et al*. 2004; Gechev *et al*. 2006). It could also result in ROS production from PSII as well as increased heat dissipation, causing photo-oxidative damage to the photosynthetic apparatus and thereby reducing photosynthesis. Similar findings have been reported for sorghum (Netondo *et al*. 2004). Hence, the relatively high photosynthesis rates and growth maintenance observed in tolerant varieties under excess Fe conditions may indicate that they were able to circumvent or limit stress-induced photo-oxidation of the photosynthetic organelles and better control ROS production. In contrast, sensitive varieties recorded relatively low photosynthetic rates and CO_2_ concentrations, suggesting a possible degradation of the thylakoid membranes (Marschner 1995) and oxidative damage. Similar results were reported for rice under Fe-toxic conditions (Stein **et al*.* 2009, 2014).

### Tissue Fe and leaf bronzing

The analysis of Fe concentration in the upper leaves, the lower leaves and the roots from all varieties indicated high accumulation of Fe in the upper leaves, especially in the sensitive variety Supa. Thus, the high leaf bronzing score for Supa could have resulted from the accumulation of high levels of Fe in the shoots. Similar findings were reported by Silveira *et al*. (2007) and Stein *et al*. (2009), who observed similar pattern of high tissue Fe in Brazilian cultivars sensitive to Fe toxicity. CK801 recorded higher levels of Fe in the upper leaves and the roots compared with IR64 but lower leaf bronzing score (3 vs 7 for IR64). Such pattern indicates the ability of CK801 to successfully deal with high amounts of intracellular Fe and maintain growth under Fe stress. However, it does not dump Fe in the old leaves (lower leaves) that are photosynthetically less active, as suggested in some reports (Audebert and Sahrawat 2000; Becker and Asch 2005; Majerus *et al*. 2007, Stein *et al*. 2009; Engel *et al*. 2012). Instead, CK801 may use other leaf mechanisms such as ROS detoxification or Fe storage in less reactive forms to tolerate high levels of Fe in the shoots (Wu *et al*. 1998; Asch *et al*. 2005; Engel 2009; Stein *et al*. 2009) and to some extent, compartment Fe in the roots.

In contrast, the other tolerant variety, Suakoko8, had the lowest Fe content across tissues. In previous studies on Fe toxicity, both types of tolerant varieties were reported and they were distinguished as tolerant includers (high Fe content, low LBS) and tolerant excluders (low Fe content, low LBS) (Becker and Asch 2005; Engel 2009; Onaga *et al*. 2016). The low tissue Fe in Suakoko8 can be attributed to the exclusion of ferrous Fe at the root level. Rice plants can achieve this by using their roots’ oxidising power, whereby molecular oxygen is released by the roots to precipitate ferrous Fe into ferric plumbaginifolia cuttings exposed to excess Fe. This could oxides and avoid a high influx of this toxic form (Fe^2+^) into explain why chlorophyll fluorescence increased with stress duration in all the varieties, although lower levels were observed in tolerant varieties compared with sensitive varieties. In addition, rising fluorescence could be related to the high dissipation of internal energy generated from the excitation of electrons as a result of damage caused by Fe toxicity on the photosynthetic apparatus. Inefficient energy transfer in the photosystems can lead to the production of ROS as a result of electron leakage from the Fe-S centres of PSI and reduced ferredoxin (Mittler *et al*. 2004; Gechev *et al*. 2006). It could also result in ROS production from PSII as well as increased heat dissipation, causing photo-oxidative damage to the photosynthetic apparatus and thereby reducing photosynthesis. Similar findings the plant tissues. Root oxidising power was proven to be an efficient tolerance mechanism in rice for growing in Fe-toxic environments (Engel *et al*. 2012; Wijerathna *et al*. 2012). However, in environments where Fe toxicity extends or occurs at late growth stages, this mechanism may be less efficient because root oxidising power decreases with age (Becker and Asch 2005).

### Biochemical responses

Starch is a major storage carbohydrate in plants. It fuels plant metabolism and growth when plants are unable to photosynthesise at night in the absence of light (Smith and Stitt 2007; Sulpice *et al*. 2009). Plants tend to store starch in their leaves for the short-term and in roots and seeds over long periods. Hence, starch stored in the leaf plays a pivotal role in the plant’s daily carbohydrate metabolism (Gibon 2004; Streb and Zeemana 2012). In accordance, in this study higher amounts of starch were recorded in the shoots of all varieties compared with those in the roots. Also, starch content was higher in the Fe-treated seedlings than in the controls for the tolerant varieties, particularly in Suakoko 8. This may have resulted from either an increased rate of starch synthesis or a decreased rate of starch breakdown in the tolerant varieties in response to Fe toxicity. Whether this is a way of anticipating starvation because Fe toxicity deeply affects photosynthesis or the result of the overall reduction of plant metabolism under Fe toxicity needs further investigation. Compared with the other varieties tested, CK801 stored a significant amount of starch in the roots under excess Fe conditions. This may have prepared it for longer starvation periods than the other varieties. According to Pantin *et al*. (2011) and Geiger *et al*. (2000), starch could serve to temporarily increase local sink strength to supply carbohydrates during periods of carbon hunger for sustained growth and metabolic processes.

In all the varieties tested in this study, decreasing levels of soluble sugars were observed in plants exposed to excess Fe. This could result from reduced photosynthesis as some studies have reported that soluble sugar fluctuations under abiotic stresses involve changes in CO_2_ assimilation and source-sink carbon partitioning (Roitsch 1999; Gupta and Kaur 2005). Another explanation could be related to the dual effect of sugars in ROS control. According to Couée *et al*. (2006), soluble sugars can be involved in, or related to, ROS-producing metabolic pathways, but they can also feed NADPH-producing metabolic pathways, such as the oxidative pentose-phosphate (OPP) pathway, which can contribute to ROS scavenging. Thus it is tempting to suppose that rice maintains a low level of soluble sugars to control ROS production, but this can also make the plants vulnerable because soluble sugars are important sources of carbon and energy that support growth.

Tolerant varieties CK801 and Suakoko 8 were observed to accumulate high starch in the shoots and roots, respectively, while maintaining high biomass. This positive correlation between starch and biomass indicates that these two varieties were able to maximise growth with increasing starch reserves, thus balancing their carbon supply and their growth. The sensitive variety Supa recorded increased starch content as well as increased soluble sugar under Fe-toxic conditions. This shows that the variety immobilised its starch reserves and sugars, and this could be attributed to the compromised growth observed in this variety, implying that assimilated carbon, which could be used to produce new photosynthetic biomass, languishes as an unproductive storage compound. However, the premature exhaustion of carbohydrate reserves observed in tolerant variety CK801 at sampling point 2 in the shoots could also be detrimental if this is not supplemented by reserves in the roots as it may trigger a starvation response in which valuable components of the cells (such as proteins) are degraded to support cellular housekeeping activities to keep the plant alive (Yu 1999).

### Conclusions

This work shows the responses of four lowland rice varieties to Fe toxicity and their ability and strategies to adjust plant growth and metabolism to overcome the stress. Differential responses were observed between the varieties and within the same tolerance group, suggesting the use of different adaptation mechanisms by the varieties. A general strategy of the tolerant varieties identified herein was sustaining nutrient absorption through continuous production of new lateral roots and balancing stomatal closure in such a way that gas exchange necessary for photosynthesis is enabled while excessive Fe flow was limited. Besides, CK801 showed Fe dilution effect by maintaining high water content in its tissues and strong shoot growth as well as late mobilisation of carbohydrates in roots and shoots. In contrast, Suakoko 8 rapidly accumulated carbon reserves as a means of surviving through the stress period. Further analysis of traits related to root Fe exclusion and ROS control are needed for a more comprehensive understanding of Fe toxicity tolerance in the potential donor varieties CK801 and Suakoko8.
